# A Proposed Association between Improving Energy Metabolism of HepG2 Cells by Plant Extracts and Increasing Their Sensitivity to Doxorubicin

**DOI:** 10.3390/toxics11020182

**Published:** 2023-02-16

**Authors:** Tamer A. Al-Shafie, Engy A. Mahrous, Mustafa Shukry, Mohammad Y. Alshahrani, Samah F. Ibrahim, Liana Fericean, Afaf Abdelkader, Mennatallah A. Ali

**Affiliations:** 1 Faculty of Dentistry, Biochemistry Department, Pharos University in Alexandria, Alexandria 21532, Egypt; 2 Faculty of Pharmacy, Pharmacognosy Department, Cairo University, Cairo 11435, Egypt; 3 Faculty of Veterinary Medicine, Department of Physiology, Kafrelsheikh University, Kafrelsheikh 33516, Egypt; 4 Research Center for Advanced Materials Science (RCAMS), King Khalid University, P.O. Box 9004, Abha 61413, Saudi Arabia; 5 Department of Clinical Laboratory Sciences, College of Applied Medical Sciences, King Khalid University, P.O. Box 61413, Abha 9088, Saudi Arabia; 6 Department of Clinical Sciences, College of Medicine, Princess Nourah bint Abdulrahman University, P.O. Box 84428, Riyadh 11671, Saudi Arabia; 7 Faculty of Agriculture, Department of Biology and Plant Protection, University of Life Sciences “King Michael I” from Timișoara, Calea Aradului 119, CUI 3487181, 300645 Timisoara, Romania; 8 Faculty of Medicine, Department of Forensic Medicine and Clinical Toxicology, Benha University, Benha 13518, Egypt; 9 Faculty of Pharmacy, Pharmacology and Therapeutics Department, Pharos University in Alexandria, Alexandria 21532, Egypt

**Keywords:** HepG2 cells sensitization, doxorubicin, cancer metabolism, asparagus, rue, green tea, avocado

## Abstract

Increasing cancer cell sensitivity to chemotherapy by amending aberrant metabolism using plant extracts represents a promising strategy to lower chemotherapy doses while retaining the same therapeutic outcome. Here, we incubated HepG2 cells with four plant extracts that were selected based on an earlier assessment of their cytotoxicity, viz asparagus, green tea, rue, and avocado, separately, before treatment with doxorubicin. MTT assays elucidated a significant decrease in doxorubicin-IC_50_ following HepG2 incubation with each extract, albeit to a variable extent. The investigated extract’s ultra-performance liquid chromatography and gas chromatography coupled with mass spectrometry (UPLC/MS and GC/MS) revealed several constituents with anticancer activity. Biochemical investigation displayed several favorable effects, including the inhibition of hypoxia-inducible factor1α (HIF1α), c-Myc, pyruvate kinase-M2 (PKM2), lactate dehydrogenase-A (LDH-A), glucose-6-phosphate dehydrogenase (G6PD), and glutaminase by asparagus and rue extracts. To less extent, HIF1α, c-Myc, PKM2, and LDH-A were partially inhibited by green tea extract, and HIF1α and glutaminase activity was inhibited by avocado oil. Undesirably, green tea extract increased glutaminase; avocado oil rose c-Myc, and both increased G6PD. In conclusion, our study confirms the potential cytotoxic effects of these plant extracts. It highlights a strong association between the ability of asparagus, green tea, rue, and avocado to sensitize HepG2 cells to doxorubicin and their power to amend cell metabolism, suggesting their use as add-on agents that might aid in clinically lowering the doxorubicin dose.

## 1. Introduction

Hepatocellular carcinoma (HCC) ranks fourth among cancer-related deaths worldwide [1]. Egypt ranks third in Africa and fifteenth in the world regarding HCC patient numbers [2]. Doxorubicin treats several common malignancies, including leukemia, lymphoma, bladder, breast, ovary, and stomach carcinoma [3]. For HCC, doxorubicin acquired particular importance when administered via the drug-eluting bead trans-arterial chemoembolization (DEB-TACE) route, allowing direct delivery of the drug into the liver, thus decreasing its systemic side effects [4]. Nevertheless, complications might arise following DEB-TACE, including nausea, vomiting, liver failure, and renal insufficiency [5]. Hence, an approach to using natural products as add-ons to doxorubicin is now emerging [6].

Plant-derived metabolites and phytochemical nutrients have long been investigated for their antiproliferative effect. More recently, some phytochemicals of different chemical classes have shown an ability to alter the microenvironment of cancer cells characterized by low pH, hypoxia, and oxidative stress [7]. Natural products have also been proven to alleviate chemotherapy-induced side effects such as gastrointestinal, hepato-, renal-, and cardiotoxicity [8]. Plant phenols, including flavonoids, stilbenes, and lignans, are potent radical scavenging molecules that alleviate oxidative stress in both in vitro and in vivo experiments [9,10].

The metabolism in cancer is reprogrammed to accommodate the high energy demands for the highly proliferating cells required for biomass buildup and tumor growth [11]. Decades ago, Otto Warburg discovered a dramatic increase in glucose uptake and lactate production by cancerous cells. Later, this phenomenon was termed the “Warburg Effect” [12,13] and is considered a significant cancer hallmark that can be exploited for designing more effective and less toxic anticancer therapies [14,15]. Several plant components have been reported to inhibit metabolic enzymes involved in the Warburg effect, such as modulating the expression of glycolysis enzymes by the flavonoids apigenin and hesperidin [16]. In the same context, we demonstrated in a recent study the ability of licorice root extract to modulate aberrant metabolism in liver cancer cells by targeting metabolism-regulating miRNAs [17]. Moreover, Guerra et al. studied the effect of phenolics, isoprenoids, and alkaloids on cancer cell metabolism and illustrated their ability to modulate various metabolic pathways in several cancer cell types [18].

Thus, the appealing approach of using natural extracts to alter the microenvironment of cancer cells could increase the cytotoxic effects of the chemotherapeutic agents and minimize their potential side effects [19,20,21]. Interestingly, a recent study proved that natural product targeting aberrant metabolism in cancer contributes to overcoming resistance while sensitizing cancer cells to chemotherapy at lower doses [22,23]. For instance, plant phenolics have been repeatedly shown to alter the cancer microenvironment, increasing cell sensitivity to anticancer agents as reported in flavonoid enriched extract of mulberry, astragalus, and Rhodiola extracts [24,25,26]. Saponins containing plants such as *Pulsatilla* and *Anemone* could alter cancer cell metabolism by regulating the MAPK pathway [27,28,29]. Recently, evidence showed that sulforaphane and glucosinolate-enriched broccoli sprout extract reversed the Warburg effect in vitro and animal models [7,30]. Finally, acetogenins from Graviola leaves extract enhanced the cytotoxicity of cisplatin in different cancer cell lines by reducing the expression of MDR1 and MAPK1 genes [31,32].

In the current study, we investigated whether the cytotoxic effect of doxorubicin could be augmented by pretreating cancer cells with a plant extract. In addition, we aimed to study the association between improving cell metabolism using a plant extract and the ability of such extracts to sensitize cancer cells to doxorubicin. To investigate such an assumption, we chose ten widely available and commonly used medicinal plants, functional foods, and condiments enriched with these aforementioned bioactive phytochemicals. Herein, we used ten herbal extracts: grapes, grapefruits, common rue, black seed, rhubarb, green tea, mustard seeds, arugula, garden asparagus, and avocado pulp. We selected these extracts to explore plant phenolics of different classes, which are abundant in grapes (anthocyanins), grapefruits and common rue (flavonoids and coumarins), black seed and rhubarb (quinones), and green tea (catechins). These plants have multiple traditional uses due to their hepatic-/neuro-/nephroprotective, antioxidant, anti-inflammatory, antiepileptic, anti-proliferative, antibacterial, and anti-atherogenic capabilities [33,34]. Both mustard seeds and arugula (family Brassicaceae) are enriched with sulfur compounds, among which glucosinolates and sulforaphane have potential cytotoxic activity. White mustard is traditionally used for muscle, joint, and chest pains [35], while arugula extract is used as an antibacterial agent [36]. Saponins are the significant constituents of garden asparagus, an edible plant with reported antitumor, antihypertensive, antioxidant, immunomodulatory, hypoglycemic, and antiepileptic properties [37]. Avocado pulp extract was also used herein as a source of acetogenins and fat-soluble vitamins and has been reported to reduce cardio-metabolic risk and possesses anticancer and antimicrobial properties [38]. Finally, we selected the four highest cytotoxic plant extracts to examine their ability to sensitize HepG2 cells to doxorubicin and interfere with the altered metabolism in HepG2 cells.

## 2. Materials and Methods

### 2.1. Plant Material

Aerial parts of common rue (*Ruta graveolens* Linn., Rutaceae), Arugula (*Eruca vesicaria* (L.) Cav., Brassicaceae) garden asparagus (*Asparagus officinalis* L., Asparagaceae) were obtained from and authenticated at the medicinal, aromatic, and poisonous plants experimental station (MAPPES), Faculty of Pharmacy, Cairo University. Seeds of black seed (*Nigella sativa* L., Ranunculaceae) and white mustard (*Sinapis alba* L., Brassicaceae) were obtained from and authenticated by Agricultural Research Center, Giza, Egypt. Avocado fruits (*Persea Americana* Mill, Lauraceae, Duke cultivar), red grapes (*Vitis vinifera* L., Vitaceae, seedless variety), and grapefruits (*Citrus paradisi* Macfad, Rutaceae) were obtained from a private garden along the Cairo-Alexandria desert road. Dr. Ayman Elkafrawy authenticated plants at the Agricultural Research Center, Giza, Egypt. Voucher specimens are kept at the Herbarium of the Department of Pharmacognosy, Faculty of Pharmacy, Cairo University. The Dried rhubarb rhizome (*Rheum officinale* Baill, Polygonaceae) and dried green tea leaves (*Camellia sinensis* (L.) Kuntze, Theaceae) were obtained from a local herbal market in Cairo, Egypt. Dr. Reem Samir Hamdy authenticated them, Lecturer of Plant Taxonomy, Botany Department, Faculty of Science, Cairo University, Giza, Egypt. Plant material was further evaluated for possible contamination by pharmacognostic examination of their morphological and microscopical characters.

### 2.2. Preparation of Plant Extracts

Tea leaves, rhubarb rhizome, white mustard, and black seeds were purchased as dried material, and aerial parts of *R. graveolens*, *E. vesicaria*, and *A. officinalis*, grapefruit peels, and grapes were dried in a vacuum-heated oven at 40 °C. All dried materials were powdered, and 50 g of each dried powder was repeatedly extracted with methanol (Adwic Co., Cairo, Egypt) until exhaustion (200 mL × 4) to obtain the methanolic extract. The fruits were peeled, thinly sliced, and dried in a vacuum oven for avocado. The dried fruits were powdered and then extracted with *n*-hexane (200 mL × 4, Adwic Co.) to obtain the fixed oil. Solvents were removed using a rotary evaporator, and the obtained residues were stored in amber-colored glass at 4 °C until use.

### 2.3. Cell Culture

Hepatocellular carcinoma cell line HepG2 (VACSERA, Egyptian company for the production of vaccines, Sera, and drugs, Giza, Egypt) was used in the current study. The cells were maintained in Dulbecco’s Modified Eagle’s Medium (DMEM) (ThermoFisher Scientific, Waltham, MA, USA). HepG2 cells were cultured in a humidified atmosphere containing 5% CO_2_ at 37 °C, and the medium was supplemented with 10% fetal bovine serum (FBS) (Sigma-Aldrich, St. Louis, MO, USA), two mM L-glutamine, 100 U/mL penicillin, and 100 µg/mL streptomycin.

### 2.4. Cytotoxicity Assay for Plant Extracts by MTT Assay

To determine the cytotoxic activity of the plant extracts or oil, 3-(4,5-dimethylthiazol-2-yl)-2,5-diphenyl-2H-Tetrazolium bromide (MTT) assay by Mosmann, 1983 was used [39]. HepG2 cell growth was assessed by treating the cells with a plant extracts concentration range of (1.562–100 or 200 µg/mL) for 48 h to detect any potential cytotoxicity. Briefly, HepG2 cells were seeded in a 96-well plate at a density of 1.0 × 10^4^ cells/well for 24 h before use. Fresh medium containing serial dilution of doxorubicin and the ten herbal extracts in DMSO was added and incubated for 48 h, followed by incubation with five mg/L MTT for four h. Control cells were exposed to DMSO alone at a concentration equal to that used in the treated cells. The supernatant was removed after centrifugation. Finally, 100 µL of DMSO was added, and the absorbance at 595 nm was determined using an ELISA microplate reader (Tecan, sunrise, Germany). The cytotoxicity assays of all plant extracts were performed, and the IC_50_ values were calculated for each using nonlinear regression in GraphPad Prism v5.0 (GraphPad Prism Inc., La Jolla, CA, USA). Based on the resultant IC_50_ values of the plant extracts, four plant extracts with the lowest IC_50_ values were selected for further experiments.

### 2.5. HepG2 Cell Treatment

Doxorubicin acts by inducing free radical-mediated oxidative damage to DNA. Therefore, synergetic treatment with antioxidant constituents can alleviate its toxicity [40]. To investigate the ability of the four selected plant extracts to sensitize HepG2 cells towards doxorubicin without interfering with the doxorubicin action, we treated HepG2 cells with two sublethal doses (IC_20_ and IC_35_) of each plant extract separately for 48 h before treatment with doxorubicin, not to hamper the doxorubicin action by the constituent’s activity of the plant extract and to allow plant extracts to exert the possible effects on cellular metabolism if any.

### 2.6. Chemical Composition Analysis

Asparagus, common rue, and green tea extracts were analyzed using ultra-performance liquid chromatography and mass spectrometry (UPLC/MS). In contrast, after derivatization, avocado oil was analyzed using gas chromatography coupled with mass spectrometry (GC/MS).

#### 2.6.1. UPLC/MS Analysis of Plant Extracts

For UPLC/MS analysis, the extracts of asparagus, common rue, and green tea were dissolved in methanol, passed through a 0.45 µm filter, and injected (7 µL) into an Agilent UPLC system Model G1316C equipped with RP-18 column (2.1 × 150 mm, particle size 1.7 μm, Waters). The column temperature was maintained at 45 °C, and separation was achieved using different binary gradients of 0.5% formic acid *v*/*v* (solvent A) and acetonitrile (solvent B) at a flow rate of 200 µL/min based on reported methods, as detailed in the supplementary data (Supplementary Figures S1–S3). The UPLC system was coupled to a triple quadrupole mass spectrometer (Waters) equipped with an Electrospray (ESI) ion source operating in negative and positive mode using the following conditions: gas temperature 325 °C, gas flow 9 L/min, nebulizer gas 45 psi, and capillary voltage at 4500 V. Masses were recorded between 100–1500 *m*/*z*. Eluted peaks were identified based on their molecular ion, elution time compared to reported literature, and fragment ions when available.

#### 2.6.2. GC/MS Analysis of Avocado Oil

Two grams of avocado oil were saponified with 5% methanolic KOH to obtain potassium salt of fatty acids. After acidification with 6N HCl, fatty acids were extracted several times with diethyl ether and later methylated using methanol/H_2_SO_4_ under reflux. After cooling, fatty acids methyl esters (FAMEs) were recovered with diethyl ether and subsequently analyzed using Trace GC Ultra system (Thermo Fisher Scientific Co., USA) equipped with Thermo TR-FAME column (70% Cyanopropyl Polysilphenylene Siloxane, 30 m × 0.25 mm i.d) with 0.25 µm film thickness using N2 at 30 mL/min and a flame ionization detector operating at a flow rate of 350 mL/min (H2). The injector temperature was set at 200 °C with an initial temperature of 140 °C, which was increased to 200 °C by five °C/min, then kept isothermal for 3 min. FAMEs were identified by comparing their relative retention times with external standards analyzed under the same conditions.

### 2.7. Cytotoxicity Assay for Doxorubicin by MTT Using Untreated and Plant Extract-Pretreated HepG2 Cells

The cytotoxicity of doxorubicin (Doxorubicin Hydrochloride, Adriblastina^®^, Pfizer Inc., USA) was determined by MTT assay (as described above) on both untreated and the plant extract-pretreated HepG2 cells. HepG2 cell growth was assessed by treating the cells with a concentration range of 0.01–200 µg/mL of doxorubicin. IC_50_ values were calculated in both cases and compared (IC50 values of all used substances were calculated using sigmoidal dose–response curve-fitting models).

### 2.8. Colorimetric/ELISA Determination of Intracellular Proteins/Enzymes

After treatment, cells were harvested with lysis buffer (RayBiotech, GA, USA, catalog no. EL-lysis), and protein/enzyme concentrations or activities were measured in cell lysates using the following commercially available kits: HIF-1α, c-Myc, and G6PD (Abcam, Cambridge, UK, catalog no. ab171577, ab277452, and ab102529, respectively), LDH-A and PKM2 (Elabscience, Wuhan, China, catalog no. E-EL-H0556 and E-EL-H6063, respectively), and glutaminase (Cohesion Biosciences, London, UK, catalog no. CAK1065) according to the manufacturer’s instructions. Protein concentrations were quantified using a BCA Protein assay kit (Abcam, Cambridge, UK, catalog no. ab102536).

### 2.9. Statistical Analysis

The statistical analysis was performed using GraphPad Prism v5.0 (GraphPad Prism Inc., La Jolla, CA, USA). MTT experiments were performed in triplicate. Data were expressed as mean ± S.D. The unpaired Student’s *t*-test was used to compare differences between the IC_50_ of doxorubicin in the untreated and the pretreated cells with either low or high doses of each herb. The differences between all untreated and treated samples were analyzed by one-way ANOVA followed by Tukey multiple comparison test. The correlation coefficients (r) between different assayed parameters were evaluated using the Pearson correlation coefficient; *p* < 0.05 was considered the significance limit for all comparisons.

## 3. Results

### 3.1. Cytotoxic Effect of Plant Extracts on HepG2 Cell

**Figure 1:**
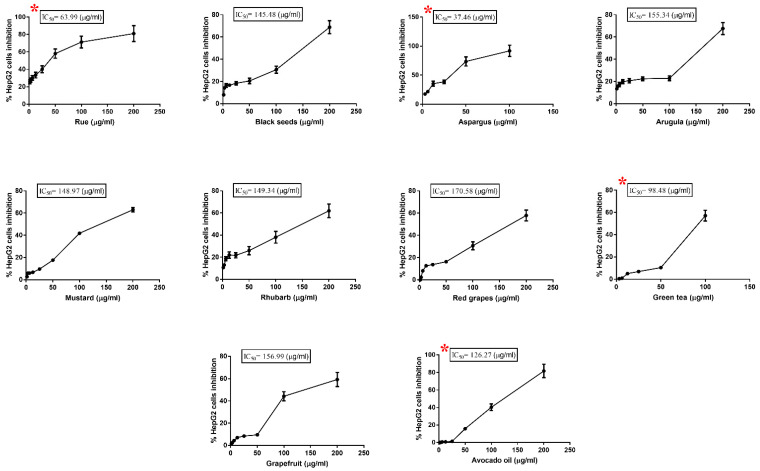
The cytotoxicity of the ten plant extracts on HepG2 cells after treatment for 48 h. Experiments were performed in triplicate. Data are presented as mean ± S.D. * Indicates the plant extract with the lowest IC_50_, which was selected for further investigation.

Our results indicated that extracts of common rue, black seeds, garden asparagus, arugula, white mustard, rhubarb, red grapes, grapefruit, green tea, and avocado oil showed variable degrees of growth inhibition of HepG2 cells, as illustrated in Figure 1.

Asparagus extract had the lowest IC_50_ at 37.46 μg/mL, while the weakest inhibition was seen with red grapes extract, with the highest IC_50_ at 170.58 μg/mL (as illustrated in Table 1).

The four extracts with the most substantial effect *viz* asparagus, rue, green tea, and avocado oil, were selected for further investigations. Phytochemical analysis to identify significant phytoconstituents of each extract was performed. Additionally, the effect of these extracts on metabolic proteins/enzyme levels and their ability to sensitize HepG2 cells toward doxorubicin was investigated. HepG2 cells were treated for 48 h with two sublethal doses (IC_35_ and IC_20_ µg/mL) of the four selected plant extracts (as illustrated in Table 2).

### 3.2. Identification of Major Phytoconstituents of the Investigated Extracts

Plant extracts contain hundreds of phytochemicals with a wide range of polarity, structural diversity, and biological activities. Biological activities exerted by certain plant extracts in biological systems usually result from many interactions between these metabolites and biological system components. Identifying these metabolites can help explain the observed biological activity and provide a basis for pinpointing the mechanism of action. Moreover, plant extract composition can vary significantly based on its geographical origin, time of collection, and extraction method. Therefore, it is often necessary to provide an analytical fingerprint of bioactive plant extracts and identify their primary metabolites.

**Figure 2:**
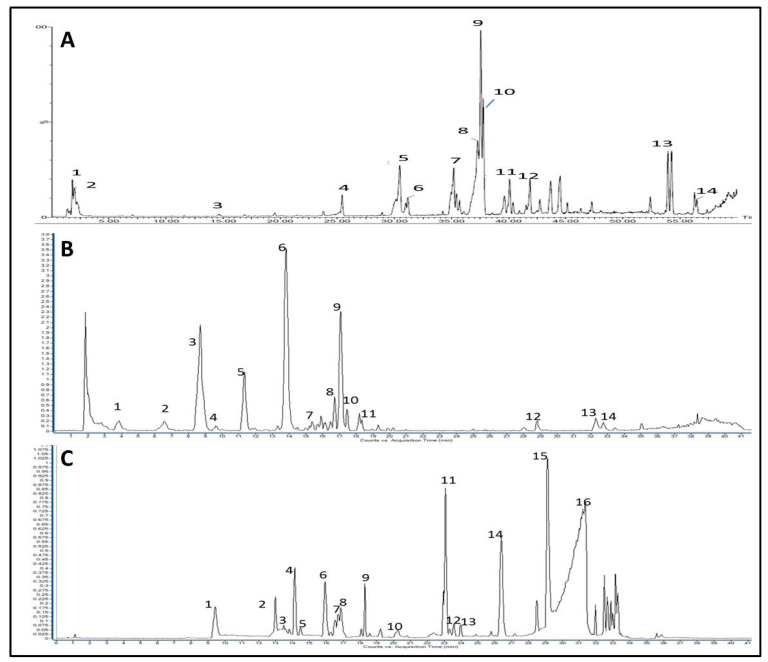
UPLC/MS chromatograms of investigated plant extract. Base peak chromatogram for the methanolic extract of (**A**) asparagus, (**B**) green tea acquired in negative ionization mode, and (**C**) rue acquired in positive ionization mode. Designated peak numbers are described in the text and Supplementary Table S1.

Ultra-performance liquid chromatography coupled to mass spectrometry (UPLC/MS) analysis of asparagus extract showed early eluted peaks identified as organic acids and phenolic acid glycosides (Figure 2A), including gluconic acid (*m*/*z* 195, peak 1), malic acid (*m*/*z* 133, peak 2), caffeic acid (*m*/*z* 179, peak 3), sinapoyl-feruloyl glucoside (*m*/*z* 723, peak 4) as summarized in Supplementary Table S1. The highest intensity peaks (retention time Rt = 36–38 min) were mainly glycerol derivatives of phenolic acids and showed characteristic loss of coumaroyl (−164 amu) and feruloyl residues (−194 amu). Accordingly, these peaks were identified as dicoumaroylglycerol (*m*/*z* 383 for [M-H]^-^, peak 8), coumaroyl feruloylglycerol (*m*/*z* 413 for [M-H]^-^, peak 9), and diferuloylglycerol (*m*/*z* 443 for [M-H]^-^, peak 10), which were previously reported in *Asparagus officinalis* (Supplementary Table S1). Several saponins were detected in the LC/MS chromatogram, albeit at a lower intensity than phenolic acid derivatives, and were eluted between 30–45 min. These saponins included asparacoside (*m*/*z* 1003, peak 5) and asparanin D (*m*/*z* 1017, peak 7). Other potential saponins were detected at *m*/*z* 943, 1001, 1017, 1019, and 1105 but could not be identified due to the limited fragmentation of their molecular ion peaks. Other phenolic compounds identified in the extract included norlignans (nyasol and methoxy nyasol, peaks 11 and 12, respectively) and phenylacetylenes (mono and tri-methoxy asparenydiol, peaks 13 and 14, respectively), Figure S1.

Meanwhile, green tea extract’s LC/MS chromatogram showed its enrichment in flavonoid compounds, especially catechin derivatives (Figure 2B). Gallic acid was the first eluted peak with relatively low intensity at Rt = 3.8 min, but its derivatives represented the major identified peaks, Figure S2. For example, the highest intensity peak (Rt = 13.9) was identified as epigallocatechin gallate (EGCG, *m*/*z* 457 for [M-H]^-^, peak 6) along with epi/catechin gallate (ECG *m*/*z* 441 for [M-H]^-^, peak 9) which were readily identifiable by the loss of gallic acid moiety (−170 amu) upon fragmentation. Six flavan-3-Ol were identified in peaks 2, 3, 4, 5, 6, 9, Supplementary Table S1. In addition to flavan-3-Ol derivatives, other flavonoids were annotated in the LC/MS chromatogram of green tea extract, including quercetin, kaempferol, and their glycosides (peaks 10–14), which were observed at a lower intensity and were eluted later in the chromatographic run, Figure 2B.

The LC/MS chromatogram of rue methanolic extract showed fewer polar constituents than observed for both asparagus and green tea extracts, Figure 2C, and eluted peaks were predominantly coumarins or alkaloids, Supplementary Table S1. Six coumarins were identified here, with the highest intensity observed for chalepin (*m*/*z* 315 for [M + H]^+^, peak 11) and chalepensin (*m*/*z* 255 for C_16_H_15_O_3_^+^, peak 14), Figure S3. In addition to coumarins, rue extract showed multiple molecular ions with an even *m*/*z* corresponding to alkaloids (peaks 1, 2, 4, 6, 7, 9, 12, 13, 15, 16). Several quinoline and acridone alkaloids were tentatively identified in Supplementary Table S1. It is worth noting that the identified quinolone alkaloids were homologs with long aliphatic chain substituents eluted late in the chromatographic run. However, MS/MS fragmentations did not provide enough fragment ions to identify these alkaloids unequivocally.

Besides the activity for the three-plant extract, fixed oil obtained from avocado pulp also showed appreciable activity. Gas chromatography analysis of fatty acids methyl esters of avocado oil identified unsaturated fatty acids as significant constituents of the FAMEs composition at 83.13% of total FAME, with oleic acid as the major fatty acid representing 53% of FAME content as outlined in Supplementary Table S2. Monounsaturated fatty acids represented 64.8% of the total fatty acids and were represented by oleic acid (53.4%), trans oleic acid (6.2%), and palmitoleic acid (5.2%).

### 3.3. Cytotoxic Effect of Doxorubicin on Pretreated and Untreated HepG2 Cells

**Figure 3:**
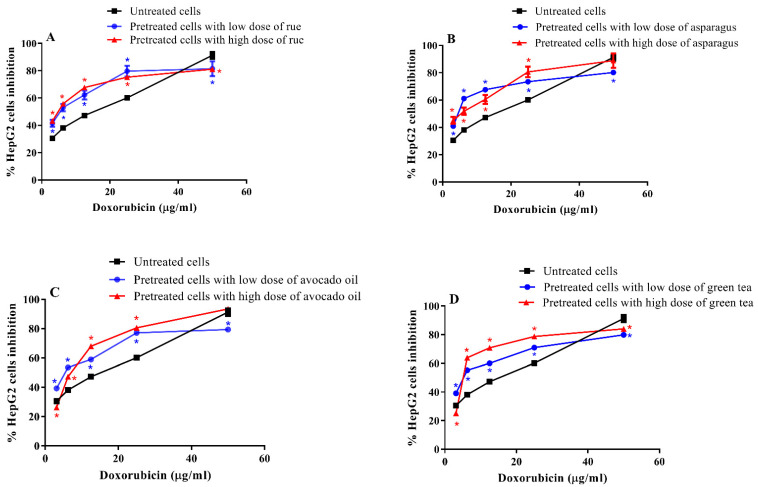
MTT assay of doxorubicin using untreated and plant extract-pretreated HepG2 cells. Doxorubicin IC_50_ was determined using untreated HepG2 cells vs. HepG2 cells pretreated with (**A**) rue in doses of 8.62 (IC_20_) and 12.08 (IC_35_) µg/mL, (**B**) asparagus in doses of 11.62 (IC_20_) and 18.08 (IC_35_) µg/mL, (**C**) avocado oil in doses of 44.56 (IC_20_) and 71.52 (IC_35_) µg/mL, or (**D**) green tea in doses of 55.58 (IC_20_) and 90.92 (IC_35_) µg/mL. Data are presented as means ± S.D. (*n* = 3) as compared with the corresponding doxorubicin concentration in the untreated HepG2 cells (*) using unpaired Student’s *t*-test at *p* < 0.05.

Our data showed a significant decrease in doxorubicin IC50 in pretreated cells compared to untreated counterparts. Doxorubicin inhibited untreated HepG2 cell growth at IC50 value equal to 7.98 µg/mL. When cells were pretreated with a high and low dose of asparagus extract, doxorubicin showed much lower IC50 equal to 3.38 and 1.73 µg/mL, respectively. To less extent, rue-pretreated HepG2 cells were sensitized towards doxorubicin represented by IC50 at 5.57 and 4.32 µg/mL for cells treated with a low and a high dose of rue extract, respectively. Additionally, doxorubicin IC50 was observed at lower values for HepG2 cells pretreated with green tea extract or avocado oil as shown in (Figure 3 and Supplementary Figure S4).

### 3.4. Effect of HepG2 Cell Treatment with Plant Extract on Metabolism-Regulating Enzymes/Proteins Level

**Figure 4:**
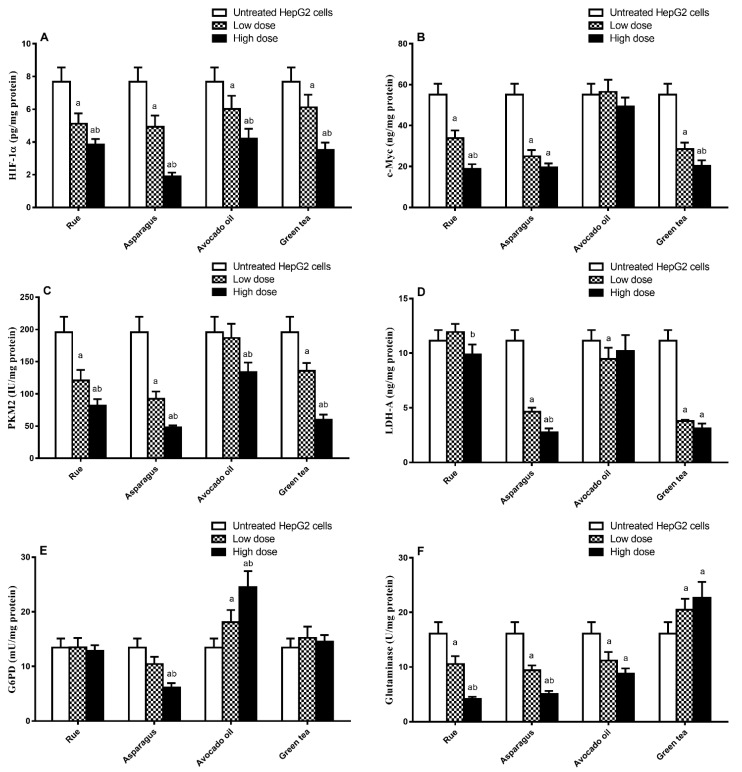
The effect of the four selected plant extract treatment on HepG2 cellular levels of (**A**) hypoxia-inducible factor1α (HIF1α), (**B**) c-Myc, (**C**) pyruvate kinase-M2 (PKM2), (**D**) lactate dehydrogenase-A (LDH-A), (**E**) glucose-6-phosphate dehydrogenase (G6PD), and (**F**) glutaminase. Data are presented as means ± S.D. (*n* = 3) as compared with untreated HepG2 cells (a) and the low dose in the corresponding group (b) using one-way ANOVA followed by Tukey post hoc test at *p* < 0.05.

Hypoxia-inducible factor1α (HIF1α) was reported to regulate rate-limiting glycolytic enzymes such as lactate dehydrogenase-A (LDH-A) and pyruvate kinase-M2 (PK-M2) [41,42,43]. Furthermore, c-Myc promotes glycolytic enzyme activation and mitochondria function suppression. Interestingly, overexpression of c-Myc was reported to influence HIF1α [44]. In our work, HepG2 cells pretreated with the investigated plant extract showed variable metabolic enzyme/protein concentrations or activities. A significant reduction in the HIF-1α level was determined in all pretreated cells compared to untreated cells (high doses induced significantly higher effects than low doses) (Figure 4A). The selected plant extracts, except for avocado oil, showed a significant decrease in c-Myc levels in low and high doses compared to untreated HepG2 cells. In contrast to rue and green tea, a non-significant difference in c-Myc was observed upon reaching both doses of asparagus extract (Figure 4B).

Moreover, PKM2 levels in HepG2 cells treated with low and high doses of rue, asparagus, and green tea extracts were significantly lower than that shown in the untreated cells (high doses induced significantly higher effects as compared to low doses). Nevertheless, only the high dose of avocado oil produced a significant reduction in PKM2 levels compared to untreated cells (Figure 4C). LDH-A levels were significantly reduced in cells treated with asparagus and green tea extracts and with a low dose of avocado oil compared to untreated cells. The high dose of rue extract showed a significant effect on LDH-A levels compared to the low dose. However, treatment with rue at both doses and the high dose of avocado oil produced a non-significant impact on LDH-A level compared to untreated cells (Figure 4D).

In cancer cells, glucose-6-phosphate dehydrogenase (G6PD), the key enzyme of the pentose-phosphate pathway (PPP) is up-regulated by hypoxia [43]. Our results show that only asparagus extract at the high dose reduced the G6PD level compared to untreated cells.

Meanwhile, rue and green tea extracts showed non-significant effects compared to untreated cells. Paradoxically, avocado fruit extract showed a significant increase in G6PD in HepG2 cells compared to untreated counterparts (Figure 4E).

The glutamine consumption rate increases in many cancers, promoted by glutaminase, reflecting the crucial role of glutaminolysis in tumor biology [45]. In our work, the glutaminase level was significantly reduced by rue and asparagus extracts treatment at both doses (high doses induced significantly higher effects compared to low doses) and avocado oil (no significant difference between high and low doses) as compared to untreated cells. In contrast, green tea significantly increased glutaminase levels compared to its effect on untreated HepG2 cells; however, a non-significant difference was detected between both doses of green tea (Figure 4F).

### 3.5. Correlation between the Reduction (%) in Doxorubicin IC_50_ and the Change in Metabolism-Regulating Enzymes/Proteins Level

To detect the association between the reduction (%) in doxorubicin IC_50_ and the metabolic proteins/enzymes level in each plant extract-pretreated HepG2 cells, a correlation study was performed between such parameters. As illustrated in Table 3, in asparagus-treated HepG2 cells, there was a significant positive correlation between the reduction percent in doxorubicin IC_50_ and HIF1α, C-Myc, PKM2, LDH-A, G6PDH, and glutaminase levels. Additionally, in rue-treated HepG2 cells, there was a significant positive correlation between doxorubicin IC_50_ and the HIF1α, C-Myc, PKM2, and glutaminase levels. In contrast, LDH-A and G6PDH levels have a non-significant correlation with doxorubicin IC_50_. Furthermore, in green tea-treated HepG2 cells, a significant positive correlation between doxorubicin IC_50_ and HIF1α, C-Myc, PKM2, and LDH-A was obtained, while G6PDH glutaminase levels have a significant negative correlation with doxorubicin IC_50_. Finally, in avocado-treated HepG2 cells, there was a significant positive correlation between doxorubicin IC_50_ and the HIF1α level, a significant negative correlation between doxorubicin IC_50_ and c-Myc, and G6PD levels. In contrast, correlations between doxorubicin IC_50_ and PKM2, LDH-A, and glutaminase levels were non-significant.

## 4. Discussion

Natural products have been widely reported for their cytotoxic effects by acting as antioxidants, anti-proliferative, and/or apoptotic agents [46]. A recent study explored the novel role of phytochemicals in overcoming resistance to chemotherapy and sensitizing cancer cells to different anticancer drugs [47]. The exploitation of such ability is an attractive target as it allows decreasing the treatment dose while retaining the same or even better therapeutic outcome and consequently minimizing various adverse effects of chemotherapy. Alongside, altered metabolism in cancer has been reported to suppress or halt the impact of several anticancer drugs [23]. In this article, we highlighted a strong association between the ability of the plant extract to sensitize HepG2 cells to doxorubicin and its ability to improve cell metabolism. We illustrated that prior treatment of HepG2 cells with either one of the four plant extracts with the highest toxicity could enhance the cytotoxic effect of doxorubicin on such cells. We explained that such an improved cytotoxic effect of doxorubicin could be attributed to interfering with the dysregulated metabolic pathways by the plant extract, which ultimately sensitizes HepG2 cells to such a chemotherapeutic drug.

The four selected plant extracts showed very different phytochemical compositions, as revealed by LC/MS analysis, which spanned other secondary metabolites.

Asparagus extract produced the most significant sensitization of Hep2G cells to doxorubicin. It showed various chemical constituents, including saponins, phenolic acids, lignans, and acetylenes. Phenolic acids such as coumaric, ferulic, and sinapic acids represent the significant constituents of the asparagus extract, and these compounds have been recognized as solid antioxidants [48]. In addition, nyasol and asparenydiol detected in asparagus extract were previously reported as moderate cytotoxic agents in different cancer cell lines [49]. Asparagus extract was also enriched in saponins, a class of compounds with the previously reported ability to inhibit HIF1α in ovarian cancer cells (ginsenosides) with eventual suppression of the Warburg effect [50,51]. Our biochemical investigation revealed that, first, asparagus extract inhibited c-Myc, a key regulator of aerobic glycolysis, by activating both PKM2 and LDH-A [52]. Second, it inhibited the glutaminolysis-regulating enzyme; glutaminase. However, glutaminase inhibition could also have occurred indirectly through inhibiting c-Myc by the extract, as c-Myc has been reported to inversely regulate glutaminase [45,53]. Third, asparagus extract treatment resulted in G6PD inhibition. Similarly, c-Myc might be involved in G6PD inhibition as it is reported to regulate such an enzyme to generate ribose 5-phosphate [52].

Common rue extract was the second primary substance in our study that showed an ability to sensitize Hep2G cells to the effect of doxorubicin. Rue is used as a condiment and herbal tea in many cultures. It has been reviewed for various biologically active constituents [54]. At least ten alkaloids were identified in the rue extract. The cytotoxic effect of rue in this study can be attributed partially to six of these alkaloids, namely the acridone type alkaloids, which exert anti-proliferative solid action [55]. Among acridone alkaloids identified in rue extract, arborinine has been reported to activate apoptosis-regulating factors Bcl-2 and Bax [56]. The most intense peak in the LC/MS chromatogram was annotated as chalepin, which was said to induce mitochondrial-mediated apoptosis in some human lung carcinoma cell lines [57]. To our knowledge, nothing was reported about the effects of standard rue extract on HepG2 cell metabolism. Our data showed that the rue extract inhibited HIF1α and c-Myc, PKM2, LDH-A, G6PD, and glutaminase. However, such an inhibitory effect on LDH-A and G6PD was statistically non-significant.

Green tea extract and avocado oil showed an appreciable, but the less, ability to sensitize HepG2 to doxorubicin compared to asparagus and rue. Both functional foods are highly reputed for their antioxidant activities attributed to catechin and unsaturated fatty acids, respectively [58,59], consistent with our phytochemical analysis results. In an exciting study by de Oliveira Júnior et al., epigallocatechin demonstrated efficacy in sensitizing tumor cells to conventional chemotherapy, including doxorubicin; however, the authors did not address the mechanism of such effect [60]. Our results showed that green tea extract inhibited HIF1α, PKM2, and LDH-A. This is consistent with Almatroodi et al., who reported that catechin in green tea decreased the expression of HIF1α in breast cancer cells and inhibited lactate production, anaerobic glycolysis, and glucose consumption in adenocarcinoma cells [61]. We further illustrated that green tea inhibited c-Myc, but undesirably it increased G6PD and glutaminase levels. Furthermore, our results showed that avocado oil failed to inhibit PKM2 and LDH-A, in agreement with Lara-Marquez et al., who reported that a lipid-rich extract of avocado oil did not influence the glycolysis rate using colorectal adenocarcinoma cells [62]. Our results illustrate some undesirable effects on metabolism induced by avocado oil, as it increased levels of c-Myc and G6PD. Nevertheless, beneficial effects of avocado oil on metabolism were also detected, as it inhibited both HIF1α and glutaminase.

## 5. Conclusions

We demonstrated that treating HepG2 cells with either asparagus, rue, green tea extracts, or avocado oil amended the metabolism and augmented the doxorubicin cytotoxic effect on such cells. Pretreatment with asparagus was the most effective in sensitizing HepG2 cells to doxorubicin and, less extent, pretreatment with rue, green tea, and avocado oil, respectively. Asparagus and rue showed the most proficiency in correcting the aberrant metabolism as they inhibited HIF1α, c-Myc, PKM2, LDH-A, G6PD, and glutaminase in HepG2 cells. With a lower ability, green tea and avocado oil also corrected the aberrant metabolism. We concluded that the ability of the plant extracts to improve doxorubicin efficacy might be partially ascribed to the amended metabolism; that is, the more amended metabolism accomplished by the extract, the more its ability to sensitize HepG2 cells. Our study recommends that other chemotherapeutic drugs should be examined to confirm whether this approach can be generalized.

## Figures and Tables

**Table 1:** IC50 of plant extracts on HepG2 cells after treatment for 48 h.

Plant Extract	IC50 (µg/mL)
Asparagus Officinalis	37.46
Ruta graveolens	63.99
Green tea	98.48
Avocado fruit	126.27
Nigella Sativa	145.48
Mustard	148.97
Rhubarb	149.34
Arugula	155.34
Grapefruit	156.99
Red grapes	170.58

**Table 2:** Comparison of doxorubicin IC50 using untreated HepG2 cells to the rue-, asparagus-, avocado-, and green tea-pretreated counterparts.

	Plant Extract Treating Dose (µg/mL)	Doxorubicin IC50 (µg/mL)	*p*-Value
**CTRL (Untreated cells)**	-	7.99 ± 0.41	
**Rue-pretreated cells**	Lo: 8.62	5.57 ± 0.35	0.012 *
	Hi: 12.08	4.32 ± 0.24	0.002 **
**Asparagus-pretreated cells**	Lo: 11.62	3.38 ± 0.19	0.001 **
	Hi: 18.08	1.73 ± 0.12	0.001 **
**Avocado-pretreated cells**	Lo: 44.56	6.82 ± 0.42	0.118
	Hi: 71.52	5.34 ± 0.31	0.007 **
**Green tea-pretreated cells**	Lo: 55.57	6.05 ± 0.32	0.021 *
	Hi: 90.92	4.80 ± 0.26	0.003 **

Lo: low dose equal to IC20, Hi: high dose equal to IC35. Values are mean ± S.E.M, and an unpaired *t*-test was used to compare two groups at a time. Non-significant *p* > 0.05. ** Significance level at *p* < 0.01.* Significance level at *p* < 0.05.

**Table 3:** Correlation between doxorubicin IC50 reduction (%) due to HepG2 cell pretreatment with four-candidate plant extracts and the decrease or increase in the metabolic proteins/enzymes level as compared to control untreated cells.

HepG2 Treatment-Induced Reduction in Doxorubicin IC50 (%)		HIF1α (ng/mL)	C-Myc (μg/mL)	PKM2 (ng/mL)	LDH-A (μmol/mL)	G6PD (mU/mL)	Glutaminase (μg/mL)
Rue-treated cells	r	0.916	0.883	0.934	0.269	0.192	0.884
	*p*-value	0.010 *	0.020 *	0.006 **	0.606	0.716	0.019 *
Asparagus-treated cells	r	0.957	0.836	0.909	0.903	0.977	0.963
	*p*-value	0.003 **	0.038 *	0.012 *	0.014 *	0.001 **	0.002 **
Avocado-treated cells	r	0.939	−0.891	0.117	0.097	−0.859	−0.374
	*p*-value	0.005 **	0.017 *	0.825	0.855	0.028 *	0.465
Green tea-treated cells	r	0.854	0.950	0.928	0.857	−0.888	−0.850
	*p*-value	0.031 *	0.004 **	0.008 **	0.029 *	0.018 *	0.32 *

Non-significant *p* > 0.05. ** Significance level at *p* < 0.01.* Significance level at *p* < 0.05.

## Data Availability

The authors confirm that the data supporting the findings of this study are available with the corresponding author.

## Supplementary Materials

The following supporting information can be downloaded at: https://www.mdpi.com/article/10.3390/toxics11020182/s1, Figure S1: Structure of major constituents identified in the extract of green asparagus; Figure S2: Structure of major constituents identified in the extract of green tea; Figure S3: Structure of major constituents identified in the extract of rue; Figure S4: Cytotoxic effect of doxorubicin on pretreated and untreated HepG2 cells; Table S1: Major Metabolites Identified in Active Plant Extracts; Table S2: Fatty Acid Composition of Avocado Oil. (Refs. [63,64,65,66,67,68,69,70,71,72] are cited in Supplementary file.)
