# Supplementary material for: A Proposed Association between Improving Energy Metabolism of HepG2 Cells by Plant Extracts and Increasing Their Sensitivity to Doxorubicin
Source: Toxics. 2023 Feb 16;11(2):182. doi: 10.3390/toxics11020182 (PMC9967676; doi:10.3390/toxics11020182)
Supplement: Supplementary file 1 [file toxics-11-00182-s001.zip › toxics-2137638-supplementary.pdf]

Chromatographic conditions for UPLC/MS analysis

The chromatographic separation of the asparagus extract followed the method described previously by Jiménez-Sánchez et al. (1). The binary gradient started at 100% A (0.5% formic acid) and 0% B (ACN), followed by a linear gradient of 20 minutes to reach 80% A and 20% B, a 10-minute gradient to 70% A and 30% B, a 10-minute gradient to 50% A and 50% B, and a 20-minute gradient to 0% A and 100% B.

The gradient used for chromatographic separation of the green tea extract was adapted from the method previously published by Savic et al. (2). The multistep gradient consisted of solvent A (0.5% formic acid in water) and solvent B (ACN), starting with 85% A and 15% B for the first 5 minutes, followed by a linear 25-minute gradient to reach 60% A and 40% B, and a further increase of solvent B to reach 5% A and 95% B at 38 minutes, followed by isocratic elution at 95% for 4 additional minutes.

The gradient used for chromatographic separation of the rue extract consisted of solvent A (0.5% formic acid in water) and solvent B (ACN). The multistep gradient started with 90% A and 10% B for the first 5 minutes, followed by a linear gradient to 50% A and 50% B for 25 min; then, an isocratic elution at the same solvent composition was used for an additional 5 min, followed by a linear 6-minute gradient to reach 100% B, which was, in turn, followed by an isocratic linear gradient to reach 100% B and to hold solvent composition for an additional 2 minutes.

Figure S1. Structure of major constituents identified in the extract of green asparagus.

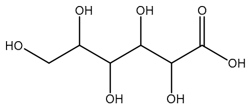

1 gluconic acid

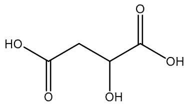

2 malic acid

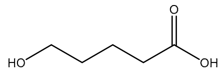

3 hydroxyvaleric acid

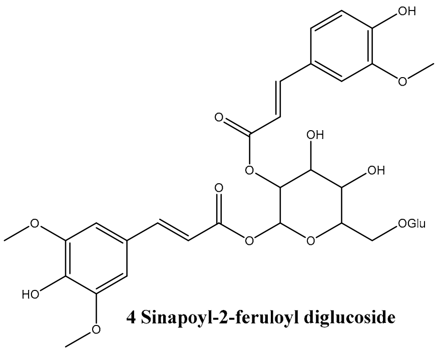

4 Sinapoyl-2-feruloyl diglucoside

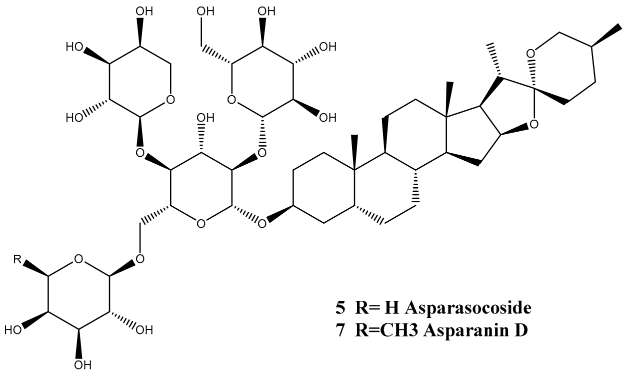

5 R= H Asparasocoside
7 R=CH3 Asparanin D

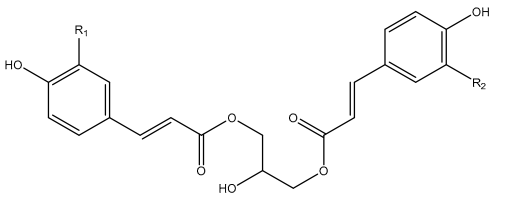

8 R1, R2 =H Dicoumaroyl glycerol
9 R1=H, R2=OCH3 Coumaroylferuloyl glycerol
10 R1, R2= OCH3 Diferulouyl glycerol

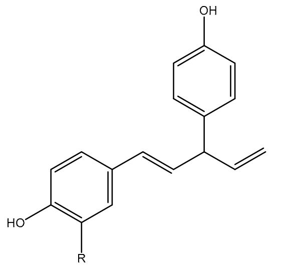

11 R= H Nyasol
12 R=OCH3 Methoxy-nyasol

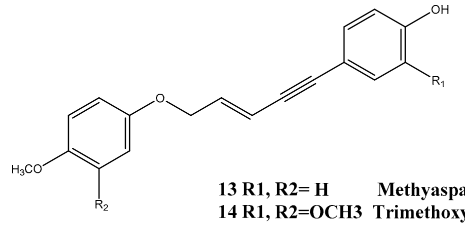

13 R1, R2= H Methyasparenydiol
14 R1, R2=OCH3 Trimethoxyasparenydiol

Structures are illustrated as a single positional isomer; however, it should be noted that positional isomers cannot be differentiated with the methodology used.

Figure S2. Structure of major constituents identified in the extract of green tea.

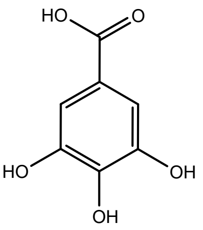

1 gallic acid

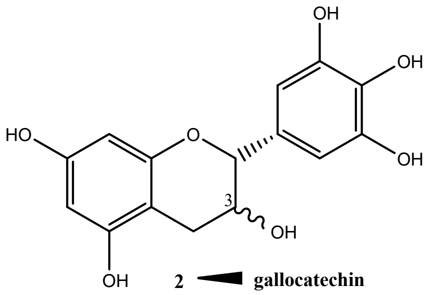

2 gallocatechin
3 epigallocatechin

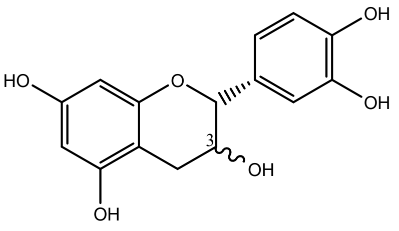

4 catechin
5 epicatechin

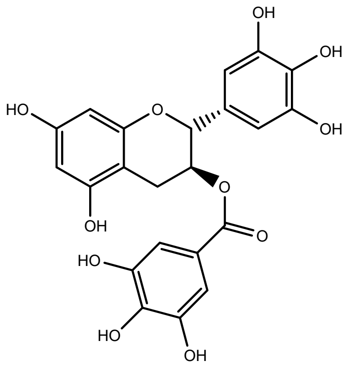

6 Epigallocatechin gallate EGCG

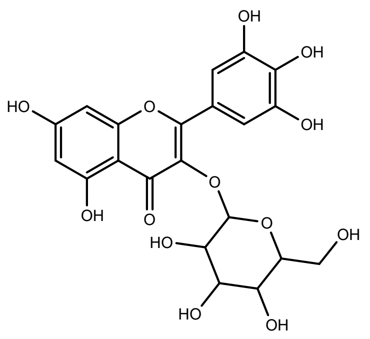

7 myricetin hexoside

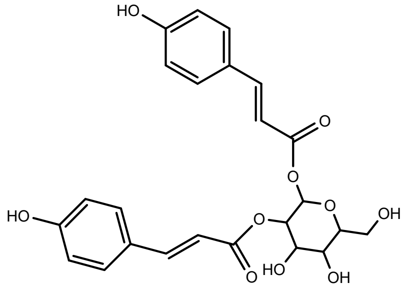

8 Dicoumaroyl hexoside

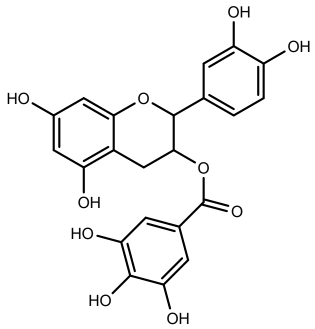

9 Epicatechin gallate ECG

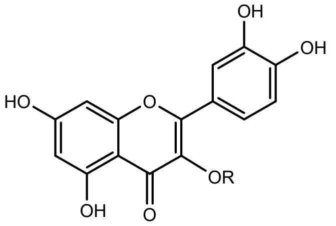

10 R=Glu-Rha rutin
11 R= hexose quercetin hexoside
12 R=H quercetin

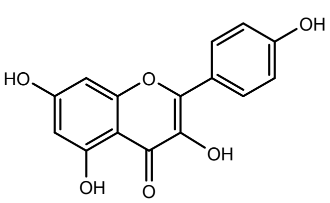

13 kaempferol

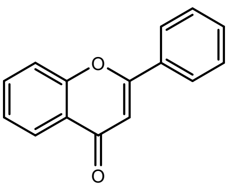

14 flavone

Figure S3. Structure of major constituents identified in the extract of rue.

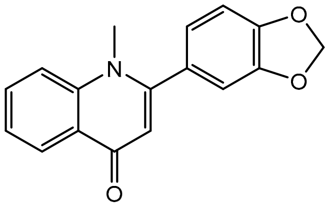

1. graveoline

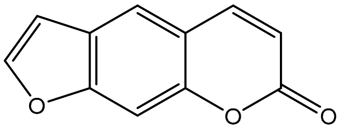

2. psoralen

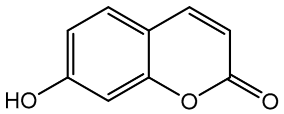

3. umbelliferone

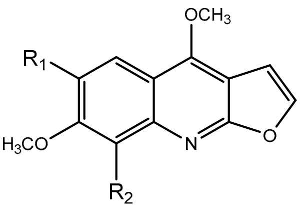

4
R1=OCH3, R2=H skimmianine
R1=H, R2=OCH3 kokusaginin

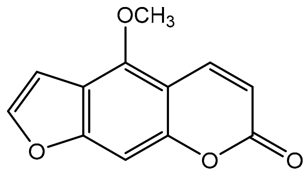

5 bergapten

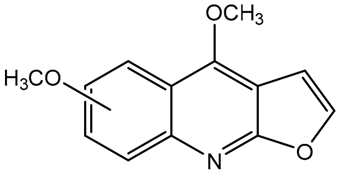

6 dictamine
7 methoxydictamine

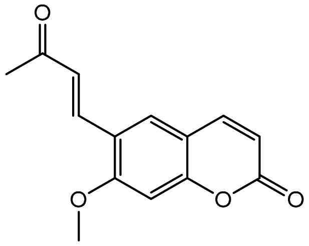

8 suberenone

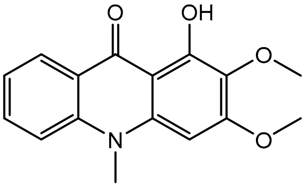

9 arborinine

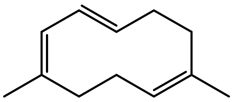

10 pregeijerene

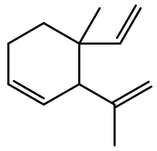

10 geijerene

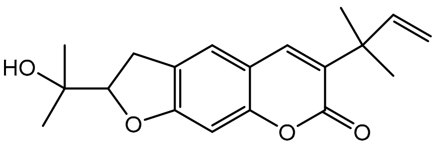

11 chalepin

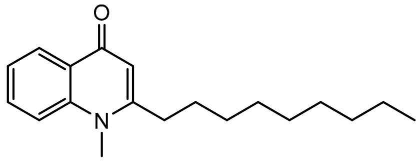

12 N-methyl, 2-nonyl-quinoline-4-one

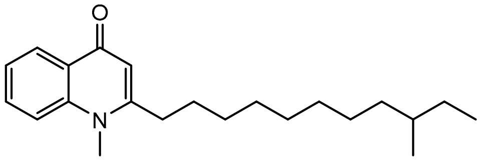

13 N-methyl, 2-(9-methylundecyl)- quinoline-4-one

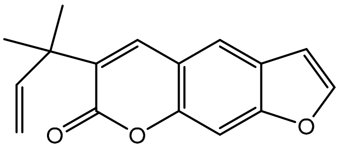

14 chalepensin

.

Figure S4. Cytotoxic effect of doxorubicin on pretreated and untreated HepG2 cells.

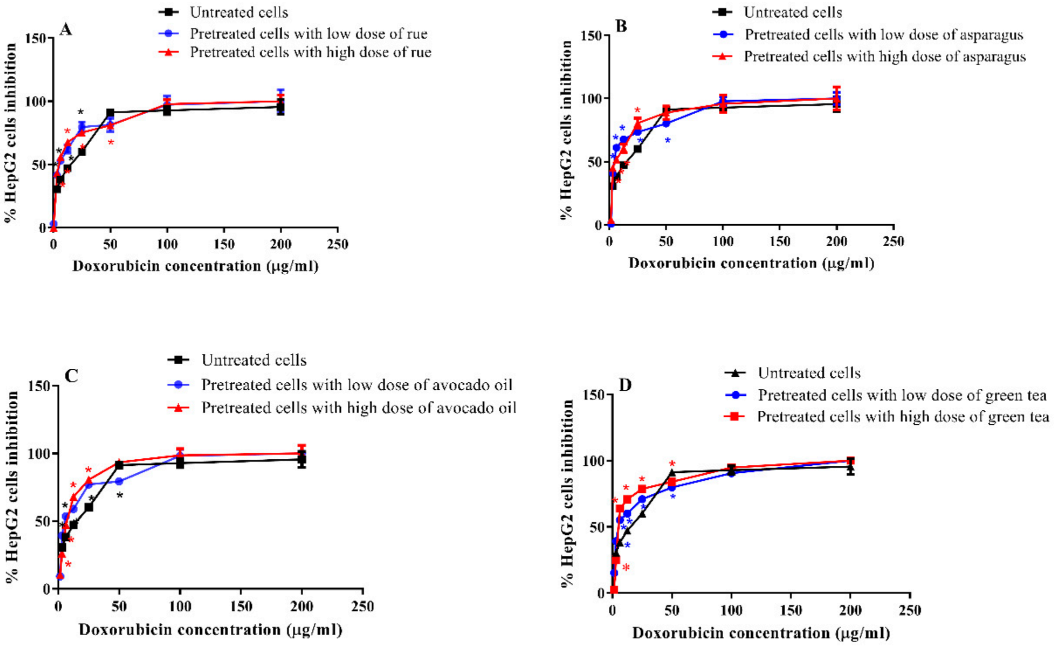

Table S1. Major metabolites identified in active plant extracts.

Plant extract	m/z	Chemical formula	Tentative identification	Chemical class	Reference
Asparagus Extract	195.2	$C_6H_{11}O_7$-	1. Gluconic acid	Organic acid	Jiménez-Sánchez et al., 2016 (1)
	133.1	$C_4H_5O_5$-	2.Malic acid	Organic acid	
	179.2	$C_9H_8O_3$-	3.Caffeic acid	Phenolic acid	
	723.4	$C_{33}H_{39}O_{18}$-	4. Sinapoyl-2-feruloyl diglucoside	Phenolic acid	
	1003.5	$C_{49}H_{79}O_{21}^-$	5.Asparacoside	Saponin	Sharma et al., 1982 (3)
	1001.5		6. Unknown	Saponin	
	1017.5	$C_{50}H_{81}O_{21}^-$	7.Asparanin D	Saponin	Zhang et al., 2004 (4)
	383.2	$C_{21}H_{19}O_7$-	8.Dicoumaroyl glycerol	Phenolic acid	Jiménez-Sánchez et al., 2016 (1)
	413.2	$C_{22}H_{21}O_8$-	9. Coumaroylferuloyl glycerol	Phenolic acid	
	443.2	$C_{23}H_{23}O_9$-	10.Diferulouyl glycerol	Phenolic acid	
	251.1	$C_{17}H_{15}O_2$-	11.Nysaol	Norlignane	Zhang et al., 2004 (4)
	281.1	$C_{18}H_{17}O_3$	12.Methoxynyasol	Nor lignan	
	279.1	$C_{18}H_{15}O_3$-	13.Methyl asparenydiol	Phenylacetylene	
	339.2	$C_{20}H_{19}O_5$-	14.Trimethoxy asparenydiol	Phenylacetylene	
Green tea Extract	169	$C_7H_5O_5$-	1.Gallic acid	Phenolic acid	Kim and Kim, 2019 (5)
	305.1	$C_{15}H_{13}O_7$-	2.Gallocatechin	Flavonoid	Savić et al., 2014 (2)
	305.1	$C_{15}H_{13}O_7$-	3.Epigallocatechin	Flavonoid	
	289.1	$C_{15}H_{13}O_6$-	4.Catechin	Flavonoid	Stöggl et al., 2004 (6)
	289.1	$C_{15}H_{13}O_6$-	5.Epicatechin	Flavonoid	
	457.1	$C_{22}H_{17}O_{11}$-	6.Epigallocatechin-gallate	Flavonoid	Kim and Kim, 2019 (5)
	479.1	$C_{21}H_{19}O_{13}$	7.Myrectin hexoside	Flavonoid	Savić et al., 2014 (2)
	471.2	$C_{24}H_{23}O_{10}$-	8.Dicoumaroyl hexose	Phenolic acid	
	441.1	$C_{22}H_{17}O_{10}$-	9. Epicatechin gallate (ECG)	Flavonoid	Kim and Kim, 2019 (5)
	609.2	$C_{27}H_{29}O_{16}$-	10.Rutin	Flavonoid	
	463.1	$C_{21}H_{19}O_{12}$-	11.Quercetin hexoside	Flavonoid	
	301.1	$C_{15}H_9O_7$-	12.Quercetin	Flavonoid	Savić et al., 2014 (2)
	285.1	$C_{15}H_9O_7$-	13.Kaempferol	Flavonoid	
	221.1	$C_{15}H_9O_2$-	14.Flavone	Flavonoid	
Rue Extract	280.1	$C_{17}H_{14}NO_3$+	1. Graveoline	Alkaloid	Ghosh et al., 2014 (7)

187.1	$C_{11}H_7O_3$+	2. Psoralen	Coumarin	Zobel and Brown, 1988 (8)
163.1	$C_9H_7O_3$+	3. Umbelliferone	Coumarin	Malik et al., 2016 (9)
259	$C_{14}H_{14}NO_4$+	4.kokusaginin/skimmianine	Alkaloid	Mancuso et al., 2015 (10)
217	$C_{12}H_9O_4$+	5.Bergapten	Coumarin	Zobel and Brown, 1988 (8)
200.1	$C_{12}H_{10}NO_2$+	6. Dictamine	Alkaloid	Kostova et al., 1999 (11)
230	$C_{13}H_{12}NO_3$	7. Methoxydictamine	Alkaloid	
245.1	$C_{14}H_{13}O_4$	8. Suberenone	Coumarin	Malik et al., 2016 (9)
286.2	$C_{16}H_{16}NO_4$	9. Arborinine	Alkaloid	Réthy et al., 2007 (12)
163.1	$C_{12}H_{19}O_3$	10. Pregeijerene/ Geijerene	Terpene	França Orlanda and Nascimento, 2015 (13)
315.2	$C_{19}H_{23}O_4$	11.Chalepin	Coumarin	Sampaio et al., 2018 (14)
286.2	$C_{19}H_{28}NO$	12. N-Methyl, 2-nonyl-quinoline-4-one	Alkaloid	Oh et al., 2014 (15)
328.3	$C_{22}H_{34}NO$	13.Methyl, 2-(9-methylundecyl)- quinoline-4-one	Alkaloid	
255.1	$C_{16}H_{15}O_3$	14. Chalepensin	Coumarin	Malik et al., 2016 (9)
338.4	ND	15. ND	Alkaloid	
368.3	ND	16. ND	Alkaloid	

Table S2. Fatty acid composition of avocado oil.

Fatty acid		Percentage of FAME
Palmitic acid	(C_{16})	16%
Palmitoleic acid	($C_{16:1}$)	5.20%
Stearic acid	(C_{18})	0.82%
Oleic acid	($C_{18:1}$)	**53.40%**
Trans oleic acid	($C_{18:1}$)	6.20%
Linoleic acid	($C_{18:2}$)	17.20%
Linolenic acid	($C_{18:3}$)	1.14%
Identified fatty acid		99.96%
Saturated fatty acids		16.82%
Unsaturated fatty acids		83.14%

References

1 Jiménez-Sánchez C, Lozano-Sánchez J, Rodríguez-Pérez C, Segura-Carretero A, Fernández-Gutiérrez A: Comprehensive, untargeted, and qualitative RP-HPLC-ESI-QTOF/MS2 metabolite profiling of green asparagus (Asparagus officinalis). *J Food Compost Anal* **46,** 78-87, 2016. doi: 10.1016/j.jfca.2015.11.004

2 Savić IM, Nikolić VD, Savić IM, Nikolić LB, Jović MD, et al.: The qualitative analysis of the green tea extract using ESI-MS method. *Savrem Tehnol* **3,** 30-37, 2014. doi: 10.5937/savteh1401030s

3 Sharma SC, Chand R, Bhatti BS, Sati OP: New oligospirostanosides and oligofurostanosides from Asparagus adscendens roots. *Planta Med* **46,** 48-51, 1982. doi: 10.1055/s-2007-970018

4 Zhang HJ, Sydara K, Tan GT, Ma C, Southavong B, et al.: Bioactive constituents from Asparagus cochinchinensis. *J Nat Prod* **67,** 194-200, 2004. doi: 10.1021/np030370b

5 Kim Y, Kim MK: *Effects of different harvesting times and oxidative fermentation methods on phytochemicals, flavors, and sensory properties of Korean teas*. In: Chemistry of Korean Foods and Beverages pp. 77-95. ACS Symposium Series, 2019.

6 Stöggl WM, Huck CW, Bonn GK: Structural elucidation of catechin and epicatechin in sorrel leaf extracts using liquid-chromatography coupled to diode array-, fluorescence-, and mass spectrometric detection. *J Sep Sci* **27,** 524-528, 2004. doi: 10.1002/jssc.200301694

7 Ghosh S, Bishayee K, Khuda-Bukhsh AR: Graveoline isolated from ethanolic extract of Ruta graveolens triggers apoptosis and autophagy in skin melanoma cells: a novel apoptosis-independent autophagic signaling pathway. *Phytother Res* **28,** 1153-1162, 2014. doi: 10.1002/ptr.5107

8 Zobel AM, Brown SA: Determination of furanocoumarins on the leaf surface of Ruta graveolens with an improved extraction technique. *J Nat Prod* **51,** 941-946, 1988. doi: 10.1021/np50059a021

9 Malik S, Moraes DFC, do Amaral FMM, Ribeiro MNS: *Ruta graveolens: Phytochemistry, Pharmacology, and Biotechnology*. In: Transgenesis and Secondary Metabolism pp. 1-28. Springer International Publishing: Cham, 2016.

10 Mancuso G, Borgonovo G, Scaglioni L, Bassoli A: Phytochemicals from Ruta graveolens activate TAS2R bitter taste receptors and TRP channels involved in gustation and nociception. *Molecules* **20,** 18907-18922, 2015. doi: 10.3390/molecules201018907

11 Kostova I, Ivanova A, Mikhova B, Klaiber I: Alkaloids and coumarins from Ruta graveolens. *Monatshefte Fur Chemie* **130,** 703-707, 1999. doi: 10.1007/PL00010251

12 Réthy B, Zupkó I, Minorics R, Hohmann J, Ocsovszki I, et al.: Investigation of cytotoxic activity on human cancer cell lines of arborinine and furanoacridones isolated from Ruta graveolens. *Planta Med* **73,** 41-48, 2007. doi: 10.1055/s-2006-951747

13 França Orlanda JF, Nascimento AR: Chemical composition and antibacterial activity of Ruta graveolens L. (Rutaceae) volatile oils, from São Luís, Maranhão, Brazil. *S Afr J Bot* **99,** 103-106, 2015. doi: 10.1016/j.sajb.2015.03.198

14 Sampaio OM, Vieira LCC, Bellete BS, King-Diaz B, Lotina-Hennsen B, et al.: Evaluation of alkaloids isolated from Ruta graveolens as photosynthesis inhibitors. *Molecules* **23,** 2693, 2018. doi: 10.3390/molecules23102693

15 Oh ET, Lee JH, Kim CS, Keum YS: Alkylquinolone alkaloid profiles in Ruta graveolens. *Biochem Syst Ecol* **57,** 384–387, 2014. doi: 10.1016/j.bse.2014.09.011
